# Supplementary figures and images for: Multi-omics analysis of parthanatos related molecular subgroup and prognostic model development in stomach adenocarcinoma
Source: PLoS One. 2025 Sep 26;20(9):e0332988. doi: 10.1371/journal.pone.0332988 (PMC12469114; doi:10.1371/journal.pone.0332988)

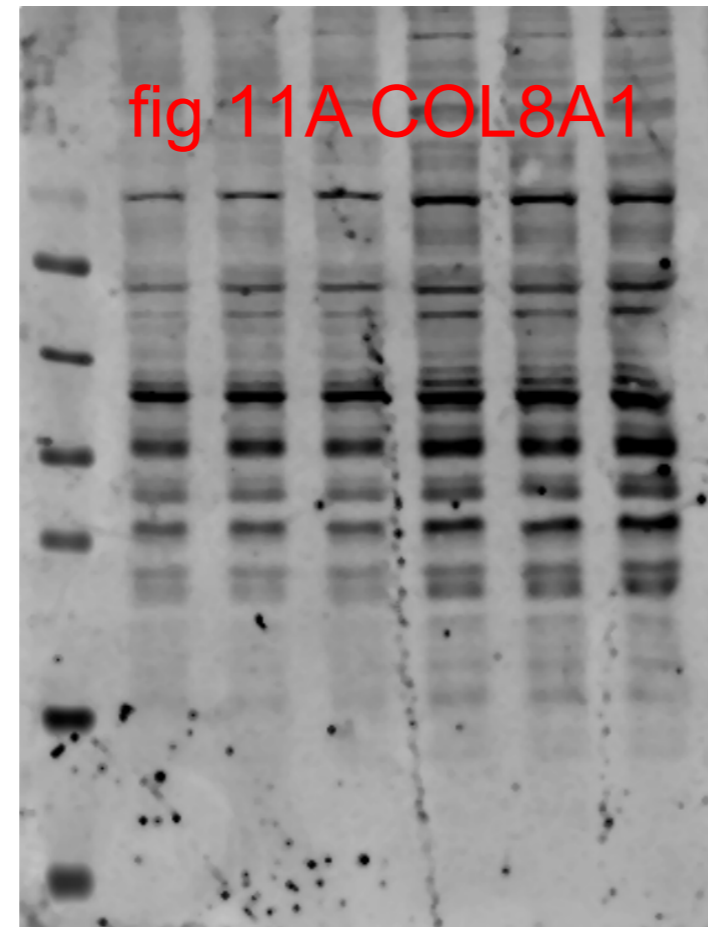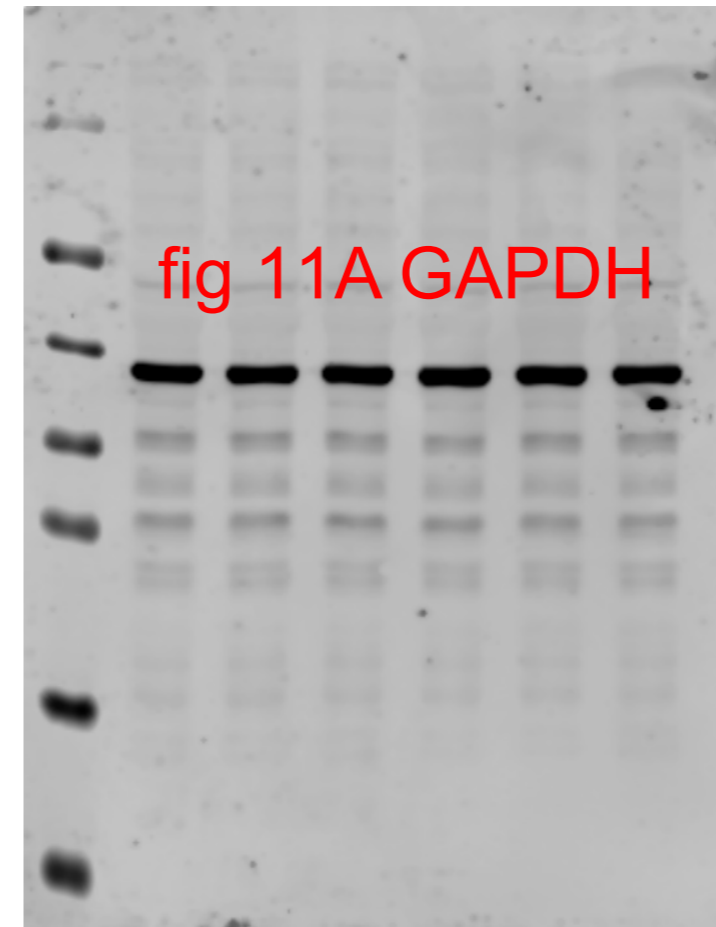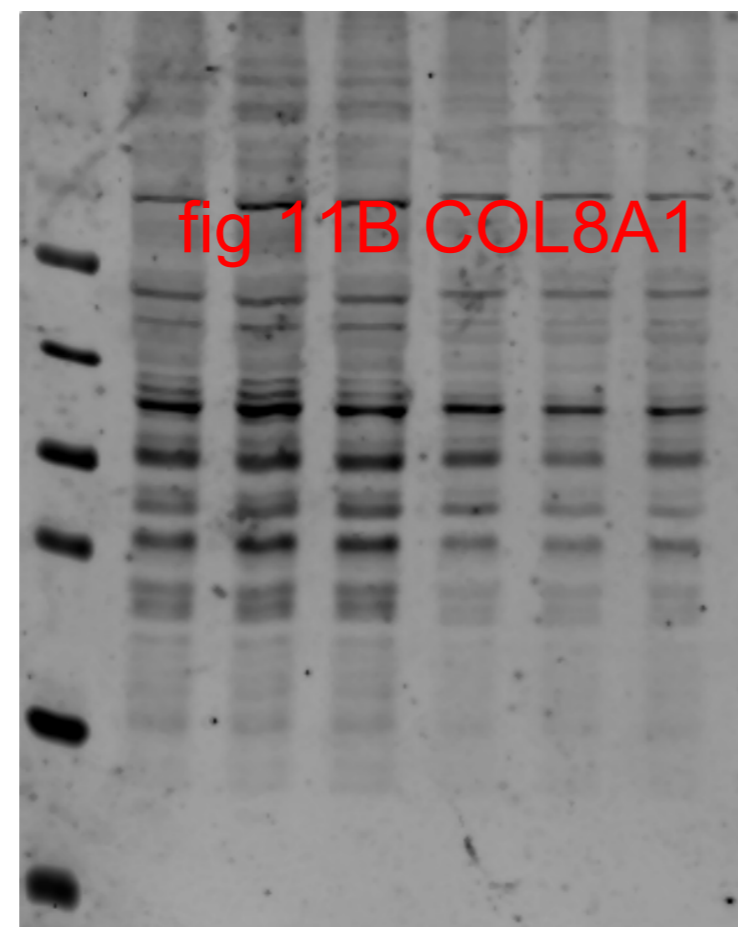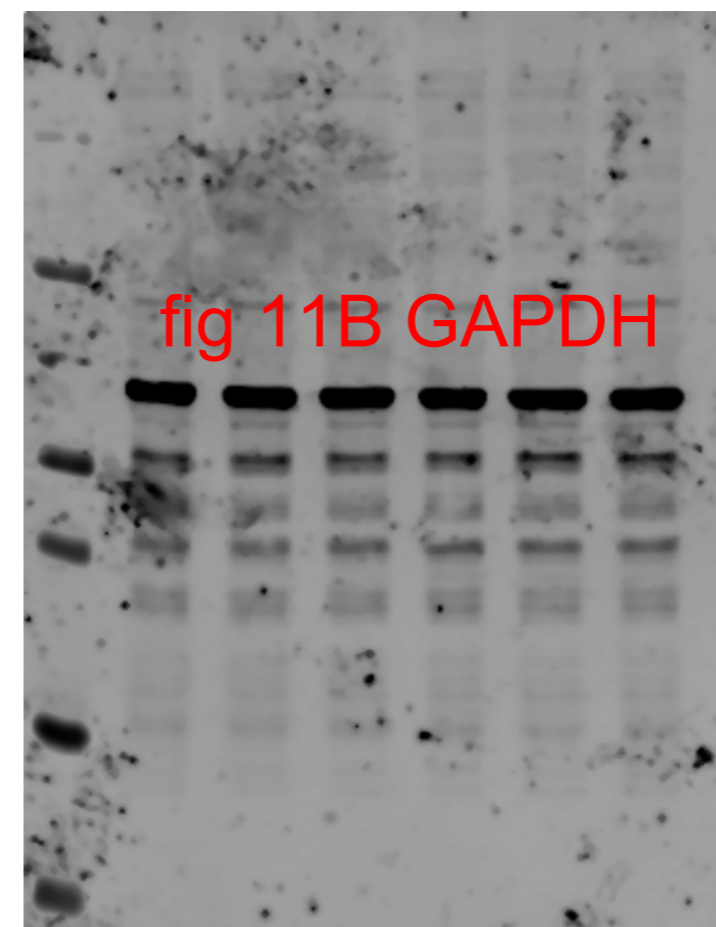

Supplement: S2 — (PDF) [file pone.0332988.s002.pdf]
