# Supplementary material for: Multi-omics analysis of parthanatos related molecular subgroup and prognostic model development in stomach adenocarcinoma
Source: PLoS One. 2025 Sep 26;20(9):e0332988. doi: 10.1371/journal.pone.0332988 (PMC12469114; doi:10.1371/journal.pone.0332988)
Supplement: Attachment — Submitted filename: Response to Reviewers.docx [file pone.0332988.s003.docx]

We sincerely appreciate the editor and all reviewers for their valuable comments and suggestions, which have significantly improved the quality and clarity of our manuscript. We have carefully considered each comment and revised the manuscript accordingly. Below, we provide a detailed point-by-point response to each comment, with our responses highlighted in bold and all modifications clearly marked in the revised manuscript.

Review Comments to the Author

Reviewer #1: The paper titled "Multi-omics Analysis of Parthanatos Related Molecular Subgroup and Prognostic Model Development in Stomach Adenocarcinoma" conducts a systematic investigation into the influence of parthanatos-associated genes in STAD. By integrating transcriptomic, genomic, and clinical data from the TCGA and GEO databases, the study defines multiple molecular subgroups that exhibit distinct parthanatos-related gene expression profiles. These clusters exhibit differences in immune infiltration status, mutational profiles, and clinical outcomes. Noteworthy is one subgroup that shows significantly elevated AIFM1 and PARP1 expression, immune evasion markers, and poor survival rates, implying a potential association between disrupted parthanatos signaling and enhanced tumor malignancy.

The authors stratify patients into high- and low-risk groups with notable survival differences using integrative analysis to create a reliable prognostic model based on parthanatos gene signatures. This model demonstrates strong predictive performance across multiple cohorts and remains independent of conventional clinical variables in multivariate analyses.The study also assesses therapeutic vulnerabilities by associating gene expression with drug response patterns, which enables the identification of promising agents for precision therapy, particularly in patients with high-risk profiles. However, several limitations exist. Since the analysis depends on retrospective datasets, its clinical applicability remains limited without further prospective confirmation. Additionally, more in-depth functional studies—both in vitro and in vivo—are necessary to clarify the mechanistic involvement of the candidate genes in parthanatos and tumor development. Future research that integrates spatial or single-cell transcriptomic technologies may enhance the understanding of the tumor microenvironment in relation to parthanatos. Collectively, the study offers meaningful insights into how parthanatos influences prognosis and treatment strategies in gastric cancer.

1. What is the current state of research on pyroptosis-associated genes in relation to STAD?

Reply:

We sincerely thank the reviewer for their insightful comments and fully understand your interest in the recent advances regarding programmed cell death mechanisms in STAD. It is important to clarify that the focus of our study is not on pyroptosis, but rather on a systematic investigation of parthanatos-related genes (PRG) in STAD, including their expression patterns, biological functions, and prognostic value.

PRG is a distinct form of programmed cell death triggered by hyperactivation of PARP1, independent of apoptosis and pyroptosis. It plays a unique role in tumor metabolic dysregulation, DNA damage response, and immune microenvironment modulation. We are aware that recent studies have suggested potential crosstalk and regulatory interactions among various forms of cell death, such as parthanatos, pyroptosis, and ferroptosis. These findings offer promising directions for future research into tumor heterogeneity.

In the revised manuscript, we have further clarified that our study focuses specifically on parthanatos and have incorporated a brief discussion in both the Introduction and Discussion sections regarding the potential interplay between parthanatos and other forms of programmed cell death, such as pyroptosis. This aims to help readers better understand the context and significance of our work.

We sincerely appreciate your attention to this important area of research, which has provided us with valuable insights and potential avenues for future exploration.

**Line 78-line 81; Line 664-667**

2. What publicly available platforms provide the transcriptomic and clinical information for STAD in this study, and how is this information processed? Including references such as Zn‐DHM Nanozymes Enhance Muscle Regeneration Through ROS Scavenging and Macrophage Polarization in Volumetric Muscle Loss Revealed by Single-Cell Profiling, Single-cell RNA sequencing and immune microenvironment analysis reveal PLOD2-driven malignant transformation in cervical cancer.

Reply:

Thank you for your valuable comments. In this study, the transcriptomic expression matrices and corresponding clinical baseline information of NC tissues and STAD tissues were obtained from two publicly accessible databases: The Cancer Genome Atlas (TCGA) and the Gene Expression Omnibus (GEO). The specific data sources and processing steps are as follows:

1: TCGA database (<https://portal.gdc.cancer.gov/>):
Transcriptomic expression data (in count format) and clinical information of STAD samples were downloaded using the R programming environment. Gene annotation and matrix processing were performed using human genome annotation files and handled via Perl scripts. Samples lacking survival information or with an overall survival (OS) of less than 30 days were excluded. After filtering, 337 STAD samples and 32 normal gastric tissue samples were included for subsequent analyses.

2: GEO database (<https://www.ncbi.nlm.nih.gov/geo/>):
Two STAD-related gene expression datasets were included: GSE84437 and GSE15459.

(1) GSE84437 (platform: GPL6947, Illumina HumanHT-12 V3.0) contains 431 STAD samples.

(2) GSE15459 (platform: GPL570, Affymetrix HG-U133 Plus 2.0) includes 182 STAD samples.
The original data were annotated and normalized using the corresponding platform annotation files via Perl scripts. Consistent with the TCGA cohort, samples lacking survival data or with OS < 30 days were excluded.

**Data integration and normalization:**
For the TCGA-STAD dataset, count data were converted into TPM (Transcripts Per Kilobase Million) format to enhance cross-sample comparability, followed by log2(TPM+1) normalization. Subsequently, TCGA and GSE84437 datasets were merged to construct the training cohort. Batch effects and normalization were corrected using the “sva” and “limma” packages in R. The GSE15459 dataset was used as an independent external validation cohort.

We have further optimized the manuscript content and cited the recommended references to improve its readability and scientific rigor.

**Line 92-line 106; line 111-line 114**

3.By what methods are DE-PRGs identified, and how is molecular subtype classification based on these genes implemented?

Reply:

We sincerely thank the reviewer for raising this insightful and professional question. The identification of differentially expressed parthanatos-related genes (DE-PRGs) and the classification process of molecular subtypes in our study were conducted as follows:

First, differential expression analysis was performed using transcriptomic data from tumor and normal tissues in the TCGA-STAD cohort, processed with the “limma” R package. The filtering criteria were set as |log2 fold change (FC)| ≥ 2 and adjusted P-value < 0.05. Genes meeting these thresholds and present in our predefined list of parthanatos-related genes (PRGs) were defined as DE-PRGs.

Next, unsupervised consensus clustering based on the expression profiles of these DE-PRGs was carried out to identify potential molecular subtypes of STAD. This was implemented using the “ConsensusClusterPlus” R package with the partitioning around medoids (PAM) algorithm. The clustering was repeated 1,000 times to ensure robustness, and we evaluated the CDF plots for cluster numbers (k) ranging from 2 to 9. The optimal number of clusters was determined based on the delta area plot and clustering stability.

To further validate the separation of identified subtypes, PCA was performed for dimensionality reduction and visualization. In addition, we compared the immune infiltration profiles and prognostic differences among the subtypes to explore their biological significance.

We sincerely appreciate your attention to the methodological details, which greatly enhances the transparency and reproducibility of our study.

**Line 119-line 122; line 125-line 132**

4. Which machine learning models are applied to create the PRG-based prognostic scoring system? Supporting references can be helpful.

Reply:

We sincerely thank the reviewer for the valuable comments. To enhance the robustness and accuracy of the prognostic scoring system based on PRGs, we systematically evaluated and compared 10 commonly used machine learning algorithms, including: CoxBoost, Enet, GBM, Lasso, plsRcox, Ridge, RSF, Stepwise Cox regression, SuperPC, and survival-SVM.

Model construction and evaluation were conducted using the corresponding R packages: “survival,” “survivalsvm,” “glmnet,” “randomForestSRC,” “CoxBoost,” “gbm,” and “Superpc.” The predictive performance of each model was assessed using the concordance index (C-index), and model stability was evaluated via 10-fold cross-validation. Based on these comparisons, the model with the highest C-index and strong generalization performance was selected to construct the final PRG-based prognostic scoring system. This model demonstrated favorable survival prediction performance in both the training cohort and external validation cohort, further supporting its clinical applicability.

In response to the reviewer’s suggestion, we have now provided a detailed description of all machine learning algorithms, analysis workflows, and performance evaluation metrics in the “Materials and Methods” section. Additionally, relevant supporting references have been added. We truly appreciate your attention to the modeling methodology, which has greatly helped us improve the clarity and completeness of this section.

**Line 180-line 185**

5. What molecular classifications are produced from the PRG-based analysis of STAD, and what correlations exist with clinical outcomes?

Reply:

We sincerely thank the reviewer for raising this important question. In our study, we performed unsupervised consensus clustering analysis based on the expression profiles of DE-PRG to classify STAD samples into three distinct molecular subtypes: Cluster A, Cluster B, and Cluster C. The clustering process was conducted using the “ConsensusClusterPlus” R package. The CDF curves and delta area plots were used to evaluate clustering stability across different values of k, and k = 3 was determined to be the optimal number of clusters.

To explore the clinical relevance of these subtypes, we conducted the following analyses:

1: Survival analysis: Kaplan-Meier survival curves showed that patients in Cluster B had significantly better OS compared to those in Cluster A and Cluster C (P < 0.001), suggesting that the subtype classification provides clear prognostic stratification.

2: Tumor immune microenvironment analysis: Using ssGSEA and the ESTIMATE algorithm, we assessed the infiltration levels of immune-related cells within the tumor microenvironment. We observed that Cluster B exhibited lower levels of immune cell infiltration, which may be associated with its favorable clinical outcomes.

We greatly appreciate your insightful suggestion. We believe that the further clarification of the molecular subtypes and their clinical correlations substantially enhances the scientific significance and translational potential of our study.

6. Immune infiltration varies significantly among different PRG molecular subtypes, which may correlate with varied responses to immunotherapy. This hypothesis is supported by studies such as PMID: 40421026 and 40406148.

Reply：

We sincerely thank the reviewer for this important comment. We fully agree that the distinct immune infiltration profiles observed among different PRG molecular subtypes may influence their responsiveness to immunotherapy. The results of our immune characterization analyses support this hypothesis: we applied multiple algorithms, including ssGSEA and ESTIMATE, to assess immune infiltration across the subtypes. Our findings revealed the following patterns:

1: Cluster A exhibited a moderate level of immune activation.

2: Cluster B showed significantly lower immune cell infiltration, indicating an “immune-cold” tumor phenotype.

3: Cluster C, despite demonstrating a high level of immune infiltration, also displayed elevated TIDE scores and reduced IPS scores, suggesting a state of immune dysfunction and immune escape.

These results further support the notion that PRG-based molecular subtypes may be closely associated with differential immunotherapy responses.

In accordance with your suggestion, we have incorporated this discussion into the revised “Discussion” section and have cited the two relevant references to strengthen the supporting evidence. We believe that these additions further enhance the scientific value of our study, particularly in the context of guiding immunotherapy strategies.

**Line 695-line 702**

7. Each gene subtype linked to PRG classification reveals unique biological characteristics and impacts patient prognosis differently. Supporting literature, such as DOI: 10.15212/bioi-2022-0008 and PMID: 27672669, explores these relationships in more detail.

Reply:

We sincerely appreciate your valuable suggestion. As requested, we have now provided a more explicit explanation regarding the correlation between PRG molecular subtyping and patient prognosis, supported by relevant literature citations. Thank you for your insightful comment, which has helped improve the clarity of our study.

**Line 685-line 695**

8. At the same time, substantial variation in tumor mutation burden and immune response is observed across PRG scoring subgroups, suggesting that PRG-based stratification could optimize immunotherapy approaches.

Reply:

We sincerely thank the reviewer for the insightful comments. Indeed, in this study, we conducted TMB analysis and immune response evaluation between the high and low PRG score groups. The results are as follows:

1: TMB analysis revealed that the low PRG score group exhibited a significantly higher TMB level, which is consistent with greater immunogenic potential.

2: Immune response prediction, as assessed by Immunophenoscore (IPS), showed that the low PRG score group had a higher predicted immunotherapy response potential, suggesting that these patients may be more likely to benefit from immune checkpoint inhibitor (ICI) treatment.

These findings support your perspective that the PRG score not only serves as a prognostic indicator but also holds promise as a predictor of immunotherapy responsiveness. The PRG-based stratification strategy may thus serve as a valuable tool for optimizing patient selection and tailoring personalized immunotherapeutic regimens in STAD.

Following your suggestion, we have incorporated the relevant comparative analyses into the “Discussion” section and further emphasized the potential clinical utility of the PRG scoring system in guiding immunotherapy decisions.

Once again, we deeply appreciate your insightful and constructive suggestions, which have greatly helped us to better articulate the link between PRG scoring and tumor immunity, and to strengthen the translational relevance of our study.

9. Although mast cells are known to be part of the STAD immune landscape, their exact role remains unclear. Consequently, future investigations with strong literature support are required to clarify their biological significance.

Reply:

We sincerely appreciate the reviewer’s forward-looking and constructive suggestion. Indeed, mast cells, as important components of the tumor immune microenvironment, have been observed to infiltrate STAD tissues to a certain extent. However, their precise role in tumor initiation and progression remains inconclusive, and no consensus has been reached in the current literature.

In our study, we evaluated immune cell infiltration using the ssGSEA algorithm, which included an analysis of mast cell activation status across different PRG-defined subtypes. The results indicated differential levels of mast cell infiltration among the subtypes; however, the direct impact of mast cells on patient prognosis could not be definitively established.

We fully agree with the reviewer that the potential role of mast cells in STAD immune regulation warrants further investigation. Future studies could leverage spatial transcriptomics, single-cell RNA sequencing, and in vivo functional assays to systematically explore their involvement in tumor immune regulation, angiogenesis, and cytokine secretion.

Following your suggestion, we have added a discussion of the uncertain role of mast cells in the “Discussion” section and highlighted possible directions for future research. Additionally, we have consulted and cited relevant literature to support this perspective.

We sincerely thank you for this valuable suggestion, which will greatly contribute to a more comprehensive understanding of the complex relationship between PRG subtypes and tumor immunity.

**Line 695-line 702**

Reviewer #2: This study conducts a multi-omics analysis of parthanatos-related genes (PRGs) in stomach adenocarcinoma (STAD), identifying molecular subtypes and constructing a PRG-based prognostic model. The integration of transcriptomic, genomic, single-cell, and functional data is well designed. The topic is relatively novel and shows clinical application potential.

However, the manuscript still has several issues regarding scientific rigor, clarity of logic, and language accuracy. Major revision is recommended before it can be considered for publication. Specific comments are as follows:

1. The manuscript contains a number of expressions influenced by Chinese syntax. A thorough language polish is needed to improve clarity, professionalism, and readability.

Reply:

We sincerely thank the reviewer for the constructive comments regarding the language and expression of the manuscript. We fully acknowledge that the original manuscript contained certain expressions influenced by Chinese linguistic conventions, which may have affected clarity and reader comprehension in some instances.

To address this issue and improve the overall quality of the manuscript, we have carefully revised the entire text to enhance its linguistic accuracy, logical flow, and academic professionalism. We believe that the revised version now presents our findings in a clearer and more concise manner, aligned with international academic standards.

Once again, we greatly appreciate your valuable suggestion, which has significantly contributed to improving the quality of our manuscript.

2. The current datasets are relatively old. The authors are encouraged to supplement with more recent publicly available datasets or their own cohort to enhance robustness and timeliness.

Reply:

We sincerely appreciate the reviewer’s valuable suggestions. We fully understand your concerns regarding the timeliness and representativeness of the data, and we agree that incorporating newer datasets can enhance the broader applicability of the study.

In this research, we selected three relatively classical publicly available stomach adenocarcinoma (STAD) cohorts—TCGA-STAD, GSE84437, and GSE15459—as the primary datasets for analysis, based on the following considerations:

1: Adequate sample size and data completeness: Compared to some recently released STAD datasets, these cohorts have relatively large sample sizes (TCGA: n = 337; GSE84437: n = 431; GSE15459: n = 182) and provide comprehensive clinical information along with long-term survival follow-up data, which are essential for robust prognostic modeling.

2: High degree of data standardization: These datasets are widely cited in gastric cancer research and have undergone stringent quality control and normalization processes, facilitating comparison and validation with other studies and improving the model’s generalizability and reproducibility.

3: Limitations of newer datasets: During our literature search, we identified some newer STAD datasets; however, many of these suffer from limitations such as small sample sizes, lack of survival information, or incomplete clinical data, making them unsuitable to meet the multifaceted requirements of molecular subtyping, immune microenvironment analysis, and prognostic modeling in our study.

Nevertheless, we highly value your suggestion and will actively seek to incorporate or integrate updated datasets in future studies to further validate and extend the applicability and clinical utility of our PRG-based classification system and prognostic scoring model.

Once again, we sincerely thank you for your attention and thoughtful advice, which has helped us carefully reconsider data selection rationale and directions for broader study application.

**Line 772-775**

3. Figure legends should be integrated into the result descriptions as per journal guidelines, rather than being placed as isolated paragraphs.

Reply:

We sincerely appreciate the reviewer’s detailed suggestions regarding the figure and text formatting. We have carefully reviewed the author guidelines of *PLOS ONE* and fully understand the requirement to integrate figure legends within the main text.

In accordance with the journal’s formatting standards, we have reorganized and revised all figure legends, embedding them into the corresponding “Results” sections. This integration allows the description of each figure to be naturally incorporated into the results narrative, thereby enhancing the coherence and readability of the manuscript.

Thank you for highlighting this important formatting issue, which has helped us further improve the quality of our submission and better align it with the journal’s publication requirements.

4. In Figure 2, the functional differences between cluster A and cluster C are not clearly discussed. A more detailed KEGG enrichment comparison between these clusters is needed.

Reply:

We sincerely thank the reviewer for the valuable comments and attention to the functional analysis details. We acknowledge that the current manuscript provides a relatively brief description of the functional differences between Cluster A and Cluster C, which may not fully capture the fundamental molecular distinctions between these subtypes.

To address this, we have supplemented Figure 2 with KEGG pathway enrichment results for both Cluster A and Cluster C. The results indicate that in the poorer prognosis Cluster C, multiple pro-tumors signaling pathways are significantly activated, including the Wnt signaling pathway, ECM-receptor interaction, and pathways in cancer. These findings reflect the highly malignant and invasive nature of this subtype. Coupled with its adverse clinical outcome, this suggests that tumors in Cluster C possess enhanced proliferative and metastatic potential.

Accordingly, we have expanded the description of the KEGG pathway enrichment analysis in the “Results” section of the revised manuscript and added comparative charts of differential pathways to enhance the completeness and persuasiveness of this part.

We greatly appreciate the reviewer’s meticulous guidance, as these additions have substantially enriched our understanding of the biological significance of the PRG-based molecular subtypes.

**Line 372-line 376**

5. The color assignments for clusters A, B and C are not consistent across figures. Please unify the color scheme to maintain visual consistency.

Reply：

We sincerely thank the reviewer for carefully pointing out the issue of inconsistent colors in the figures. We have thoroughly reviewed all figures involving Clusters A, B, and C and confirmed that some legends did indeed use inconsistent color schemes. To ensure visual coherence and improve the reader’s experience, we have standardized the color schemes across all relevant figures and reorganized the images accordingly. We believe these revisions have enhanced the professionalism and overall aesthetics of the manuscript. We greatly appreciate your valuable suggestion, which has helped us improve the manuscript’s details.

6. This is a highlight of the study but is underdeveloped. The authors are advised to incorporate deeper cell–cell communication analyses (e.g., using CellChat) to better characterize TAM heterogeneity and immune-stromal interactions.

Reply:

We sincerely appreciate the reviewer’s recognition of the key focus of our study and your valuable suggestions. We fully acknowledge that cell–cell communication within the tumor microenvironment, especially the heterogeneity of tumor-associated macrophages (TAMs) and their complex interactions with immune and stromal cells, plays a critical role in tumor progression and immune evasion.

Based on your recommendation, we have supplemented our analysis by applying the CellChat tool to systematically explore cell–cell communication networks using single-cell transcriptomic data. The results of this CellChat analysis have been added to the “Results” section of the revised manuscript, and the analytical workflow and parameter settings are described in detail in the Methods section.

We thank the reviewer again for these constructive comments, which have helped us deepen the scope of our study and enhance the novelty and scientific value of our manuscript.

**Line 233-line 248; line 590-line 606, Figure 10**

7. Although COL8A1 knockdown was experimentally validated, the underlying mechanism is not sufficiently discussed. Please explore its potential involvement in EMT, PI3K/AKT pathway, or ECM remodeling, and consider validating these axes.

Reply：

We sincerely thank the reviewer for recognizing our experimental work and providing valuable suggestions regarding the mechanistic exploration. We fully understand the importance of elucidating the specific role of COL8A1 in gastric cancer, particularly its association with key pathways such as EMT, the PI3K/AKT signaling cascade, and extracellular matrix (ECM) remodeling. Such insights will enrich the depth of our study and guide future clinical applications.

Currently, we are actively conducting related mechanistic studies, including functional validation in cell models and pathway analyses, aiming to comprehensively reveal the regulatory network and molecular mechanisms of COL8A1. However, as these mechanistic experiments are still ongoing and the relevant data are not yet mature, we are unable to fully report them in the present manuscript. We acknowledge this as a limitation of our current study.

We have candidly addressed this limitation in the “Discussion” section and clearly outlined our plans and directions for further in-depth mechanistic investigations. In the future, we intend to supplement the molecular mechanism data to systematically clarify the biological functions and potential therapeutic value of COL8A1 in STAD.

Once again, we greatly appreciate the reviewer’s professional advice, which has greatly motivated us to continue deepening our research and advancing scientific understanding in this field.

**Line 746-line 757**

8. The use of “PARG” to represent “parthanatos-related genes” is potentially misleading, as PARG typically refers to poly(ADP-ribose) glycohydrolase. Please revise and unify the terminology throughout the manuscript.

Reply：

We sincerely thank the reviewer for the detailed and professional reminder. We fully recognize that the abbreviation “PARG” has broad and potentially ambiguous meanings within the biological field. To avoid any misunderstanding and enhance terminological accuracy, we have replaced all instances of “PARG” in the manuscript with “PRG” (an abbreviation for parthanatos-related genes) and ensured consistent usage of this term throughout the entire text.

This revision covers the title, abstract, main text, figure legends, and all related expressions, thereby eliminating confusion and improving the manuscript’s professionalism and clarity.

We appreciate your valuable suggestion, which has helped us refine the manuscript’s details and make the content more rigorous and standardized.

9. Although parthanatos is a relatively novel concept in cancer biology, the authors should further clarify its unique value in gastric cancer and elaborate on how their findings differ from previous studies.

Reply：

We sincerely thank the reviewer for the valuable suggestion. We fully acknowledge that parthanatos, as an emerging form of programmed cell death, remains at an early stage of understanding in tumor research, with relatively limited studies in the context of gastric cancer.

Our study is the first to systematically construct a molecular classification and prognostic model for gastric cancer based on parthanatos-related genes, revealing the potential role of the parthanatos mechanism in tumor heterogeneity, immune microenvironment regulation, and patient prognosis in STAD. This differs significantly from prior studies focused on poly(ADP-ribose) metabolism or apoptosis-related pathways and fills a gap in the characterization of parthanatos features in gastric cancer.

Furthermore, through multi-omics data integration combined with analyses of immune infiltration, tumor mutation burden, and immunotherapy response indicators, we comprehensively elucidate the unique biological functions and clinical application potential of parthanatos-related genes in gastric cancer.

We have supplemented the “Introduction” and “Discussion” sections of the revised manuscript to emphasize the novelty of our work and its distinctions from previous studies, highlighting the unique value of parthanatos in precision therapy and risk assessment for gastric cancer.

Once again, we greatly appreciate your insightful comments, which have greatly contributed to enhancing the scientific depth of our manuscript.

**Line 647-line 663**

10. Ensure that figure numbering and font sizes are consistent across all figures to improve overall visual presentation.

Reply:

We sincerely thank the reviewer for the constructive suggestions regarding the image presentation details. We have carefully reviewed and standardized the formatting and font settings across all figures to enhance overall visual consistency and professionalism.

Specifically, the adjustments include:

1: All figure legend numbers are uniformly set to 24 pt, while all texts, annotations, and labels within the figures are set to 12 pt.

2: Considering differences in the number of groups and layout structures across figures, we applied differential aspect ratio adjustments (width and height) in some images to ensure complete display of grouped content and maintain high resolution. This may cause slight variations in the perceived relative font size among different figures; however, the actual font sizes remain consistent. This approach balances image clarity with content readability.

We have made every effort to maintain a consistent visual style without compromising the clarity or accuracy of the figure content.

Thank you again for your valuable suggestion, which has helped us improve the quality and overall presentation standards of the figures in our manuscript.

Also,the references cited in this article are not sufficient, and there is a lack of in-depth comparative discussion. Background and methodology also require further literature support. Some related research should be cited:

1. Role of natural products in tumor therapy from basic research and clinical perspectives, 10.15212/AMM-2023-0050

2. Identifying the key genes of Epstein-Barr virus-regulated tumour immune microenvironment of gastric carcinomas，10.1111/cpr.13373

3. Single-cell RNA sequencing reveals immune cell dysfunction in the peripheral blood of patients with highly aggressive gastric cancer，10.1111/cpr.13591

4. Macrophage differentiation in enhancing hematopoietic function of ribonucleic acid for injection II via multi-omics analysis, 10.15212/AMM-2024-0001

5. Prospective study and validation of early warning marker discovery based on integrating multi-omics analysis in severe burn patients with sepsis, 10.1093/burnst/tkac050

Reply:

Thank you very much for your helpful recommendations. We have carefully reviewed all the references you suggested and have incorporated them appropriately into the revised manuscript. In addition, we have expanded the Introduction and Methods sections to provide a more comprehensive background and clearer description of our analytical procedures. These revisions have significantly improved the clarity and academic depth of our work, and we sincerely appreciate your valuable input.
