# Supplementary material for: Multi-omics analysis of parthanatos related molecular subgroup and prognostic model development in stomach adenocarcinoma
Source: PLoS One. 2025 Sep 26;20(9):e0332988. doi: 10.1371/journal.pone.0332988 (PMC12469114; doi:10.1371/journal.pone.0332988)
Supplement: Attachment — Submitted filename: Response_to_Reviewers_auresp_2.docx [file pone.0332988.s004.docx]

We sincerely appreciate the editor and all reviewers for their valuable comments and suggestions, which have significantly improved the quality and clarity of our manuscript. We have carefully considered each comment and revised the manuscript accordingly. Below, we provide a detailed point-by-point response to each comment, with our responses highlighted in bold and all modifications clearly marked in the revised manuscript.

Review Comments to the Author

Please use the space provided to explain your answers to the questions above. You may also include additional comments for the author, including concerns about dual publication, research ethics, or publication ethics. (Please upload your review as an attachment if it exceeds 20,000 characters)

Reviewer #1: (No Response)

Reviewer #3: The author has made detailed revisions to the comments of the previous reviewer, thus resolving many issues of imprecise expression. However, after reading the manuscript, the only thing I feel inappropriate is that the author's conclusion is too lengthy. The conclusion should be a highly condensed and summarized version of the research findings, rather than a secondary repetition of the entire research process. It is recommended that the author streamline the conclusion.

Reply：

We sincerely thank you for taking the time to review our manuscript and for providing your valuable feedback. We highly appreciate your comment regarding the "overly lengthy conclusion section." Upon careful review, we agree that the original conclusion was not sufficiently concise and partially repeated content from earlier sections of the manuscript.

In accordance with your suggestion, we have revised and streamlined the conclusion to focus more clearly on the core findings and key innovations of our study. Redundant descriptions of the research process have been removed to enhance clarity, coherence, and summarization.

Once again, we are truly grateful for your insightful comments and recognition of our work. Your suggestions have significantly contributed to improving the quality of the manuscript, and we sincerely hope that the revised version meets your expectations.

**Line 78-Line 85; line 773-line 778**

Reviewer #4: This study, for the first time through integrated multi-omics analysis, systematically elaborates on the molecular subtypes, prognostic value, and immune microenvironment regulatory mechanisms of parthanatos-related genes (PRGs) in stomach adenocarcinoma (STAD), and experimentally verifies the cancer-promoting function of the key gene COL8A1. The research design is rigorous with advanced methods (covering transcriptome, single-cell sequencing, machine learning, etc.) and has clear potential for clinical translation. The manuscript has been substantially revised according to the reviewers' comments, but there are still some issues that need further improvement.
Main Advantages
Innovation and Clinical Significance
(1) For the first time, a molecular typing system (Cluster A/B/C) of PRGs in STAD is established, revealing its associations with prognosis, immune infiltration, and treatment response, which provides a new perspective for precision therapy.
(2) The PRG prognostic model (StepCox + Enet) constructed based on 10 machine learning algorithms shows robustness in both the training set and the validation set (GSE15459) (with outstanding C-index) and is independent of clinicopathological factors.
(3) Experimental verification of the function of COL8A1 in promoting the proliferation/migration of STAD cells supports its potential as a therapeutic target.
Methodological Rigor
(1) The integration of multi-omics data is comprehensive (TCGA, GEO cohorts) with strict quality control (excluding samples with OS < 30 days).
(2) Single-cell analysis (GSE163558) combined with CellChat reveals the cell interaction network in the tumor microenvironment, enhancing the reliability of the conclusions.
(3) The immune microenvironment assessment is diversified (ESTIMATE, ssGSEA, TIDE, IPS), corroborating the association between PRG subtypes and immune therapy response.

Key Issues and Improvement Suggestions

Insufficient Depth of Molecular Mechanisms (Key Defect)
Issue: The cancer-promoting mechanism of COL8A1 only stays at the phenotypic level (proliferation/migration), lacking verification at the pathway level (such as EMT, PI3K/AKT). The discussion mentions that "PI3K/AKT induces COL8A1-mediated ECM remodeling" (Line 748), but there is no experimental support.
Suggestion: Supplement WB verification of EMT markers (E-cadherin/Vimentin) or PI3K/AKT pathway proteins after COL8A1 knockout; or clearly list mechanism research as a future direction and emphasize this limitation in the discussion.

Reply：

Thank you for your thoughtful review and valuable suggestions regarding our study. You rightly pointed out that the current manuscript lacks in-depth mechanistic validation of how COL8A1 promotes tumor progression, particularly in terms of experimental evidence involving EMT markers (such as E-cadherin and Vimentin) and the PI3K/AKT signaling pathway.

We fully agree with your insightful comments. Due to experimental limitations and manuscript length constraints, we were unable to include additional Western blot analyses in the current version. However, we have clearly acknowledged this limitation in the Discussion section and have elaborated on our plan to conduct further mechanistic investigations—specifically, to validate the involvement of EMT processes and the PI3K/AKT pathway—in future studies.

We believe that these additions will enhance the scientific rigor and logical coherence of the manuscript. Once again, we sincerely appreciate your constructive feedback and recognition of our work.

**Line 760-Line 772**

Contradictions in Immune Microenvironment Analysis
Issue: Cluster C shows high immune infiltration but the worst prognosis, and the authors attribute it to "immune dysfunction" (Line 696-698), but there is a lack of key evidence (such as T cell exhaustion markers, proportion of immunosuppressive cells).
Suggestion: Supplement the expression heatmap of exhaustion markers such as PD-1/CTLA-4 and LAG-3 in Cluster C. Analyze the differences in infiltration of Treg/MDSC between high and low PRG score groups (the existing ssGSEA only provides an overview of 23 cell types).

Reply：

We sincerely thank the reviewer for the professional and constructive comments. In response to the concern regarding the lack of sufficient evidence in the immune microenvironment analysis, we have further supplemented the expression profiles of T cell exhaustion markers, including PD-1, CTLA-4, and LAG-3, across different subtypes (including Cluster C). These results have been incorporated into the revised manuscript.

Additionally, we have analyzed and presented the infiltration levels of immunosuppressive cells such as regulatory T cells (Tregs) and MDSCs in the high and low PRG score groups, as shown in Figure 8E. The relevant differences are also highlighted in the figure.

We believe that these additions provide stronger support for the potential association between "immune dysfunction" and poor prognosis. Once again, we truly appreciate your insightful guidance on our work.

**Line 405-Line 410; line 421-line 422, Line 540-line 544, Figure 3J-M, Figure 8E**
